# 4-Bromobenzoyl 4-bromobenzoate monohydrate

**DOI:** 10.1107/S1600536811022264

**Published:** 2011-06-18

**Authors:** Bonell Schmitt, Thomas Gerber, Eric Hosten, Richard Betz

**Affiliations:** a Nelson Mandela Metropolitan University, Summerstrand Campus, Department of Chemistry, University Way, Summerstrand, PO Box 77000, Port Elizabeth 6031, South Africa

## Abstract

In the title compound, C_14_H_8_Br_2_O_3_·H_2_O, the organic and water mol­ecules both have crystallographically imposed *C*_s_ symmetry. The dihedral angle between the aromatic rings is 45.76 (11)°. In the crystal structure, inter­molecular C—H⋯O and O—H⋯O hydrogen bonds link the mol­ecules into chains parallel to the *a* axis. No π–π stacking inter­actions are observed in the crystal structure.

## Related literature

For the crystal structure of anhydrous *para*-bromo­benzoic acid anhydride, see: McCammon & Trotter (1964 ▶); Duesler *et al.* (1981 ▶). For the use of chelate ligands in coordination chemistry, see: Gade (1998 ▶). For details of graph-set analysis of hydrogen bonds, see: Etter *et al.* (1990 ▶); Bernstein *et al.* (1995 ▶).
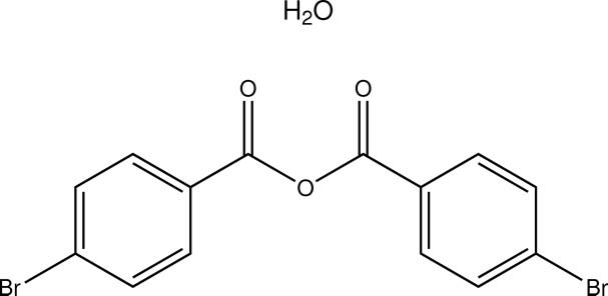


## Experimental

### 

#### Crystal data

C_14_H_8_Br_2_O_3_·H_2_O
*M* *_r_* = 402.04
Orthorhombic, 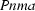

*a* = 12.6118 (3) Å
*b* = 28.2378 (7) Å
*c* = 3.8898 (1) Å
*V* = 1385.27 (6) Å^3^
*Z* = 4
Mo *K*α radiation
μ = 5.86 mm^−1^
*T* = 200 K
0.35 × 0.12 × 0.05 mm

#### Data collection

Bruker APEXII CCD diffractometer
Absorption correction: multi-scan (*SADABS*; Bruker, 2008 ▶) *T*_min_ = 0.825, *T*_max_ = 1.000
11620 measured reflections
1728 independent reflections
1519 reflections with *I* > 2σ(*I*)
*R*_int_ = 0.024

#### Refinement

*R*[*F* ^2^ > 2σ(*F* ^2^)] = 0.043
*wR*(*F* ^2^) = 0.093
*S* = 1.30
1728 reflections
98 parameters
2 restraints
H atoms treated by a mixture of independent and constrained refinement
Δρ_max_ = 0.69 e Å^−3^
Δρ_min_ = −0.62 e Å^−3^

### 

Data collection: *APEX2* (Bruker, 2010 ▶); cell refinement: *SAINT* (Bruker, 2010 ▶); data reduction: *SAINT*; program(s) used to solve structure: *SHELXS97* (Sheldrick, 2008 ▶); program(s) used to refine structure: *SHELXL97* (Sheldrick, 2008 ▶); molecular graphics: *ORTEP-3* (Farrugia, 1997 ▶); software used to prepare material for publication: *SHELXL97* and *PLATON* (Spek, 2009 ▶).

## Supplementary Material

Crystal structure: contains datablock(s) I, global. DOI: 10.1107/S1600536811022264/rz2605sup1.cif

Structure factors: contains datablock(s) I. DOI: 10.1107/S1600536811022264/rz2605Isup2.hkl

Supplementary material file. DOI: 10.1107/S1600536811022264/rz2605Isup3.cdx

Supplementary material file. DOI: 10.1107/S1600536811022264/rz2605Isup4.cml

Additional supplementary materials:  crystallographic information; 3D view; checkCIF report

## Figures and Tables

**Table 1:** Hydrogen-bond geometry (Å, °)

*D*—H⋯*A*	*D*—H	H⋯*A*	*D*⋯*A*	*D*—H⋯*A*
O90—H901⋯O2^i^	0.84 (1)	2.24 (2)	3.043 (6)	161 (5)
C3—H3⋯O2^ii^	0.95	2.58	3.211 (5)	124

Symmetry codes: (i) 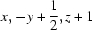; (ii) 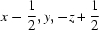.

**Fig. 1.**
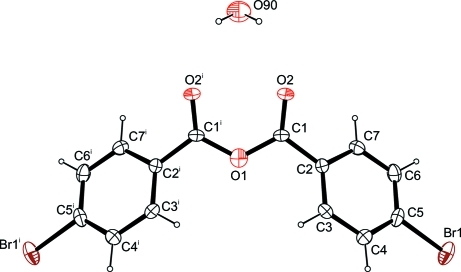
The molecular structure of the title compound, with anisotropic displacement ellipsoids drawn at 50% probability level. Symmetry code: (i) x, -y + 1/2, z.

**Fig. 2.**
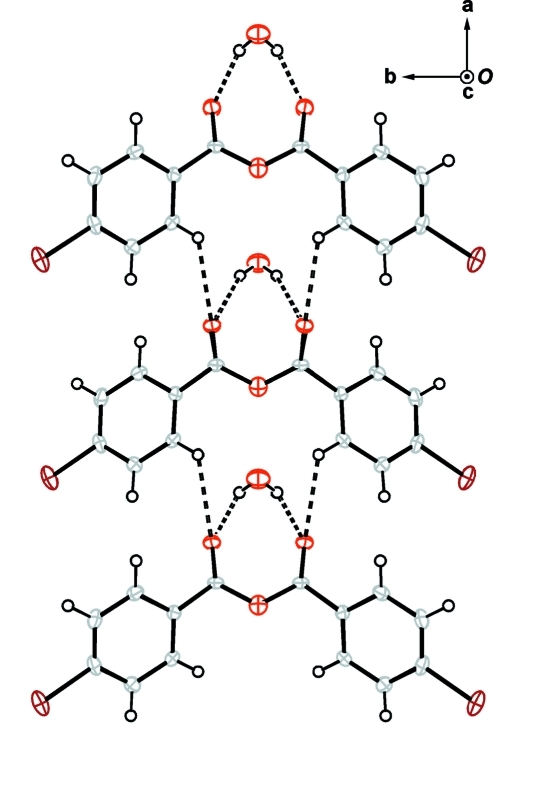
Intermolecular contacts, viewed along [001]. Displacement ellipsoids are drawn at 50% probability level.

**Fig. 3.**
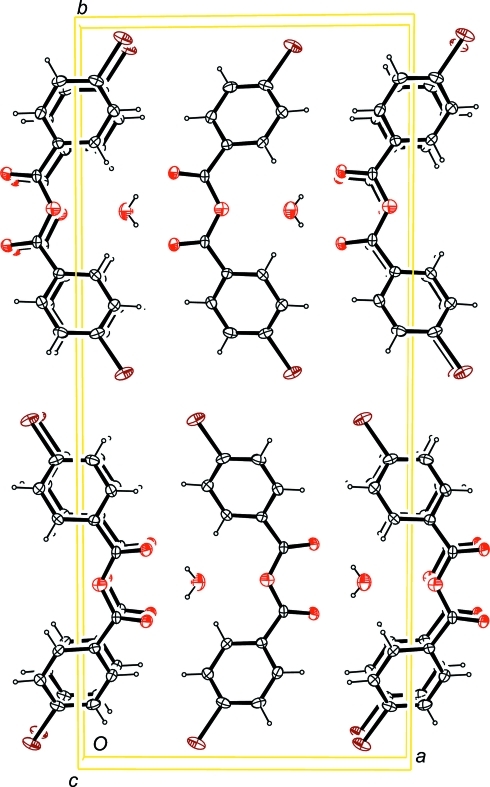
Molecular packing of the title compound, viewed along [001]. Displacement ellipsoids drawn at 50% probability level.

## supplementary crystallographic information

### Comment

Chelate ligands have found widespread use in coordination chemistry due to the enhanced thermodynamic stability of resultant coordination compounds in relation to coordination compounds exclusively applying comparable monodentate ligands (Gade 1998). Combining different sets of donor atoms in one chelate ligand molecule, a probe for testing and accomodating metal centers of different Lewis acidities is at hand. In our efforts to synthesize a chelate ligand featuring a set of oxygen, sulfur and nitrogen as possible donor atoms, a crystalline reaction product was obtained whose crystal structure analysis revealed the unintentional synthesis of the hydrated anhydride of *para*-bromobenzoic acid. The crystal structure of the anhydrous anhydride is apparent in the literature (McCammon & Trotter, 1964; Duesler *et al.*, 1981).

The asymmetric unit comprises half of the organic molecule and half of the molecule of crystal water. The least-squares planes defined by the atoms of both aromatic moieties intersect at an angle of 45.76 (11)° (Fig. 1). In the crystal structure, intermolecular C—H···O and O—-H···O hydrogen bonds can be observed (Table 1). The carbonylic O atoms of the anhydride serve as twofold acceptors for the hydrogen bonds originating from the water molecule as well as the H atom of an adjacent molecule bonded to the C atom in *ortho* position to the carboxylic acid functionality. A description of the C—H···O hydrogen bonds in terms of graph-set analysis (Etter *et al.*, 1990; Bernstein *et al.*, 1995) necessitates a *R*^2^_2_(14) descriptor on the unitary level. In total, the moieties of the crystal structure are connected to chains parallel to the crystallographic *a* axis (Fig. 2). No π–π stacking interaction is observed. The packing of the compound in the crystal structure is shown in Fig. 3.

### Experimental

The compound was prepared upon reacting 4-bromobenzyl chloride (2.5 mmol) with potassium thiocyanate (2.5 mmol) and dipyridin-2-ylamine (2.5 mmol) in refluxing acetone (15 ml) for two hours. Crystals suitable for the X-ray diffraction study were obtained upon free evaporation of the reaction mixture.

### Refinement

Carbon-bound H atoms were placed in calculated positions (C—H = 0.95 Å) and were included in the refinement in the riding model approximation, with *U*_iso_(H) set to 1.2*U*_eq_(C). The H atom of the water molecule was located on a difference Fourier map and refined using a *DFIX* instruction (*d*_O—H_ set to 0.85 Å), with *U*_iso_(H) set to 1.5*U*_eq_(O).

### Crystal data

**Table:**

C_14_H_8_Br_2_O_3_·H_2_O	*F*(000) = 784
*M**_r_* = 402.04	*D*_x_ = 1.928 Mg m^−^^3^
Orthorhombic, *P**n**m**a*	Mo *K*α radiation, λ = 0.71073 Å
Hall symbol: -P 2ac 2n	Cell parameters from 4619 reflections
*a* = 12.6118 (3) Å	θ = 2.9–28.1°
*b* = 28.2378 (7) Å	µ = 5.86 mm^−^^1^
*c* = 3.8898 (1) Å	*T* = 200 K
*V* = 1385.27 (6) Å^3^	Needle, yellow
*Z* = 4	0.35 × 0.12 × 0.05 mm

### Data collection

**Table:**

Bruker APEXII CCD diffractometer	1728 independent reflections
Radiation source: fine-focus sealed tube	1519 reflections with *I* > 2σ(*I*)
graphite	*R*_int_ = 0.024
φ and ω scans	θ_max_ = 28.3°, θ_min_ = 2.9°
Absorption correction: multi-scan (*SADABS*; Bruker, 2008)	*h* = −16→16
*T*_min_ = 0.825, *T*_max_ = 1.000	*k* = −26→37
11620 measured reflections	*l* = −3→5

### Refinement

**Table:**

Refinement on *F*^2^	Primary atom site location: structure-invariant direct methods
Least-squares matrix: full	Secondary atom site location: difference Fourier map
*R*[*F*^2^ > 2σ(*F*^2^)] = 0.043	Hydrogen site location: inferred from neighbouring sites
*wR*(*F*^2^) = 0.093	H atoms treated by a mixture of independent and constrained refinement
*S* = 1.30	*w* = 1/[σ^2^(*F*_o_^2^) + (0.0068*P*)^2^ + 7.342*P*] where *P* = (*F*_o_^2^ + 2*F*_c_^2^)/3
1728 reflections	(Δ/σ)_max_ < 0.001
98 parameters	Δρ_max_ = 0.69 e Å^−^^3^
2 restraints	Δρ_min_ = −0.62 e Å^−^^3^

### Fractional atomic coordinates and isotropic or equivalent isotropic displacement parameters (Å^2^)

**Table:**

	*x*	*y*	*z*	*U*_iso_*/*U*_eq_	
Br1	−0.14551 (4)	0.030824 (15)	−0.21724 (12)	0.03488 (15)	
O1	0.0688 (4)	0.2500	0.0995 (14)	0.0340 (11)	
O2	0.2098 (2)	0.20237 (11)	0.2371 (14)	0.0530 (12)	
C1	0.1190 (3)	0.20645 (15)	0.1450 (12)	0.0244 (9)	
C2	0.0521 (3)	0.16413 (13)	0.0633 (11)	0.0208 (8)	
C3	−0.0548 (3)	0.16199 (14)	0.1493 (11)	0.0224 (8)	
H3	−0.0880	0.1880	0.2604	0.027*	
C4	−0.1125 (3)	0.12164 (14)	0.0718 (11)	0.0228 (8)	
H4	−0.1854	0.1197	0.1316	0.027*	
C5	−0.0637 (3)	0.08430 (14)	−0.0924 (11)	0.0225 (8)	
C6	0.0432 (3)	0.08532 (14)	−0.1753 (12)	0.0257 (9)	
H6	0.0761	0.0592	−0.2863	0.031*	
C7	0.1007 (3)	0.12551 (14)	−0.0919 (12)	0.0240 (9)	
H7	0.1744	0.1267	−0.1414	0.029*	
O90	0.3607 (4)	0.2500	0.7343 (14)	0.0354 (10)	
H901	0.329 (4)	0.2690 (15)	0.864 (12)	0.053*	

### Atomic displacement parameters (Å^2^)

**Table:**

	*U*^11^	*U*^22^	*U*^33^	*U*^12^	*U*^13^	*U*^23^
Br1	0.0490 (3)	0.0237 (2)	0.0319 (2)	−0.0150 (2)	−0.0019 (2)	−0.0033 (2)
O1	0.031 (2)	0.027 (2)	0.044 (3)	0.000	−0.003 (2)	0.000
O2	0.0195 (14)	0.0242 (15)	0.115 (4)	0.0002 (13)	−0.028 (2)	0.006 (2)
C1	0.0119 (17)	0.0224 (19)	0.039 (2)	−0.0004 (14)	−0.0018 (16)	0.0024 (18)
C2	0.0185 (18)	0.0157 (18)	0.028 (2)	0.0000 (15)	−0.0027 (16)	0.0032 (16)
C3	0.0190 (18)	0.0183 (18)	0.030 (2)	0.0043 (15)	−0.0010 (17)	−0.0004 (16)
C4	0.0207 (18)	0.0215 (19)	0.026 (2)	−0.0021 (15)	0.0018 (17)	0.0019 (17)
C5	0.032 (2)	0.0164 (18)	0.020 (2)	−0.0068 (16)	−0.0036 (17)	0.0024 (16)
C6	0.032 (2)	0.0169 (18)	0.028 (2)	0.0044 (16)	0.0029 (18)	−0.0031 (17)
C7	0.0181 (18)	0.0231 (19)	0.031 (2)	0.0034 (15)	0.0022 (17)	0.0018 (18)
O90	0.029 (2)	0.044 (3)	0.033 (3)	0.000	0.006 (2)	0.000

### Geometric parameters (Å, °)

**Table:**

Br1—C5	1.893 (4)	C3—H3	0.9500
O1—C1^i^	1.395 (4)	C4—C5	1.378 (6)
O1—C1	1.395 (4)	C4—H4	0.9500
O2—C1	1.205 (5)	C5—C6	1.387 (6)
C1—C2	1.497 (5)	C6—C7	1.385 (6)
C2—C7	1.389 (6)	C6—H6	0.9500
C2—C3	1.390 (5)	C7—H7	0.9500
C3—C4	1.385 (5)	O90—H901	0.840 (14)
			
C1^i^—O1—C1	123.7 (5)	C5—C4—H4	120.1
O2—C1—O1	123.6 (4)	C3—C4—H4	120.1
O2—C1—C2	121.5 (4)	C4—C5—C6	121.8 (4)
O1—C1—C2	114.9 (3)	C4—C5—Br1	119.1 (3)
C7—C2—C3	119.9 (4)	C6—C5—Br1	119.2 (3)
C7—C2—C1	118.0 (4)	C7—C6—C5	118.1 (4)
C3—C2—C1	122.0 (4)	C7—C6—H6	120.9
C4—C3—C2	119.5 (4)	C5—C6—H6	120.9
C4—C3—H3	120.2	C6—C7—C2	120.9 (4)
C2—C3—H3	120.2	C6—C7—H7	119.5
C5—C4—C3	119.7 (4)	C2—C7—H7	119.5
			
C1^i^—O1—C1—O2	2.1 (10)	C2—C3—C4—C5	−0.6 (6)
C1^i^—O1—C1—C2	−176.7 (4)	C3—C4—C5—C6	1.8 (7)
O2—C1—C2—C7	−35.8 (7)	C3—C4—C5—Br1	−176.8 (3)
O1—C1—C2—C7	143.1 (4)	C4—C5—C6—C7	−0.7 (7)
O2—C1—C2—C3	141.8 (5)	Br1—C5—C6—C7	177.8 (3)
O1—C1—C2—C3	−39.4 (6)	C5—C6—C7—C2	−1.5 (7)
C7—C2—C3—C4	−1.5 (6)	C3—C2—C7—C6	2.6 (7)
C1—C2—C3—C4	−179.0 (4)	C1—C2—C7—C6	−179.8 (4)

Symmetry codes: (i) *x*, −*y*+1/2, *z*.

### Hydrogen-bond geometry (Å, °)

**Table:**

*D*—H···*A*	*D*—H	H···*A*	*D*···*A*	*D*—H···*A*
O90—H901···O2^ii^	0.84 (1)	2.24 (2)	3.043 (6)	161 (5)
C3—H3···O2^iii^	0.95	2.58	3.211 (5)	124

Symmetry codes: (ii) *x*, −*y*+1/2, *z*+1; (iii) *x*−1/2, *y*, −*z*+1/2.
